# Upper limb practice with a dynamic hand orthosis to improve arm and hand function in people after stroke: a feasibility study

**DOI:** 10.1186/s40814-023-01353-8

**Published:** 2023-07-27

**Authors:** Yih Wong, Louise Ada, Grethe Månum, Birgitta Langhammer

**Affiliations:** 1 Research Department, Sunnaas Rehabilitation Hospital, Bjørnemyr, Norway; 2 Institute of Clinical Medicine, Faculty of Medicine, University of Oslo, Oslo, Norway; 3 Faculty of Medicine and Health, University of Sydney, Sydney, Australia; 4 Department of Physiotherapy, Faculty of Health Sciences, OsloMet-Oslo Metropolitan University, Postboks 4, St. Olavs Plass, 0130 Oslo, Norway

**Keywords:** Stroke, Upper extremity, Home training, Dynamic orthotic device, Rehabilitation

## Abstract

**Background:**

Dynamic hand orthosis may help upper limb recovery by keeping the wrist and hand in an optimal position while executing a grasp. Our aim was to investigate the feasibility of combining a dynamic hand orthosis with task-oriented upper limb practice after stroke.

**Method:**

Fifteen adult stroke survivors were recruited in a single-group, pre-post intervention study. They received 12 weeks of task-oriented upper limb training with a dynamic hand orthosis with 3 weeks supervised at a community rehabilitation unit followed by 9 weeks unsupervised at home. Feasibility was determined by recruitment (proportion of eligible/enrolled and enrolled/retained participants), intervention (adherence, acceptability, and safety) and measurement (time taken to collect outcomes and proportion of participants where all measures were collected). Clinical outcomes were measured at baseline (Week 0), end of Week 3 and Week 12.

**Results:**

Fifteen (46%) of eligible volunteers were enrolled in the study. Eight (53%) of those enrolled completed the 12-week intervention. Eighty eight percent were satisfied or very satisfied with the dynamic hand orthosis. Clinical measures were collected for all participants at baseline and in all those who completed the intervention but often took over one hour to complete. At 12 weeks, participants had improved by 7 points out of 57 (95% CI 2 to 13) on the ARAT and by 8 points out of 66 (95% CI 0 to 15) on the FMA-UE.

**Conclusion:**

The intervention appears to be feasible in terms of acceptability and safety, while recruitment and measurement need further consideration. The magnitude of the clinical outcomes suggests that the intervention has a potential to improve both upper limb activity and impairment, and this study provides useful information for the design of a pilot randomized trial.

**Trial registration:**

ClinicalTrials.gov Identifier: NCT03396939.

## Key messages

- The intervention appears to be feasible in terms of acceptability and safety and the magnitude of the clinical outcomes suggests that the intervention has a potential to improve both upper limb activity and impairment.
- However, the trial was not feasible in terms of recruitment and measurement and changes in the eligibility criteria, as well as delivering the intervention across multiple sites or as wholly home-based may alleviate these problems.

## Background

Stoke remains a major cause of disability throughout the world. Feigin et al. estimated that in 2010 there were 33·million stroke cases worldwide [1]. Despite advances in stroke care and rehabilitation, there is a significant long-term decline in functional status after stroke [1–3]. Two thirds of stroke survivors have reduced arm and hand function in the acute stage [4] and 50% of these will still have problems later on [4, 5]. The consequences are grave for these individuals, since reduced arm and hand function may lead to increased dependence on assistance from another person in activities of daily living (ADL), restricted social participation, low quality of life, anxiety and poor well-being [6–8]. Therefore, there is a need for improvement in upper limb rehabilitation [1, 4].

Current upper limb practice after stroke has generally been aimed at counteracting the learned non-use and achieving independent use of the hand in ADL [9, 10]. One systematic review recommends repetitive, task-oriented practice for improving upper limb function [9]. However, impaired finger extension often hinders active practice. Dynamic hand orthoses can be used as a training tool since they keep the wrist and hand in an optimal position for grasping and manipulation of objects. These easy-to-use devices may encourage mass repetition as well as enabling individuals to actively participate in ADL. Interventions featuring a combination of task-oriented practice and assistive devices are gaining considerable attention [11]. They may be a solution for many stroke survivors since self-directed home-based practice is now more common due to limited resources in the community [12].

However, there are few reports of effectiveness of dynamic hand orthoses during stroke recovery [13, 14]. A recent systematic review by Alexander et al. found a positive effect for the use of dynamic hand orthoses in terms of upper limb activity (MD 6 points out of 57, 95% CI 0–12, *p* = 0.04 on the ARAT). However, the effect was based on two small studies (*n* = 29) [14]. Therefore, we designed a task-oriented program incorporating the use of a dynamic hand orthosis. The aim of this initial study was to investigate the feasibility of combining a dynamic hand orthosis with task-oriented upper limb practice in people with poor hand and arm function after stroke.

## Method

### Design

**Fig. 1:**
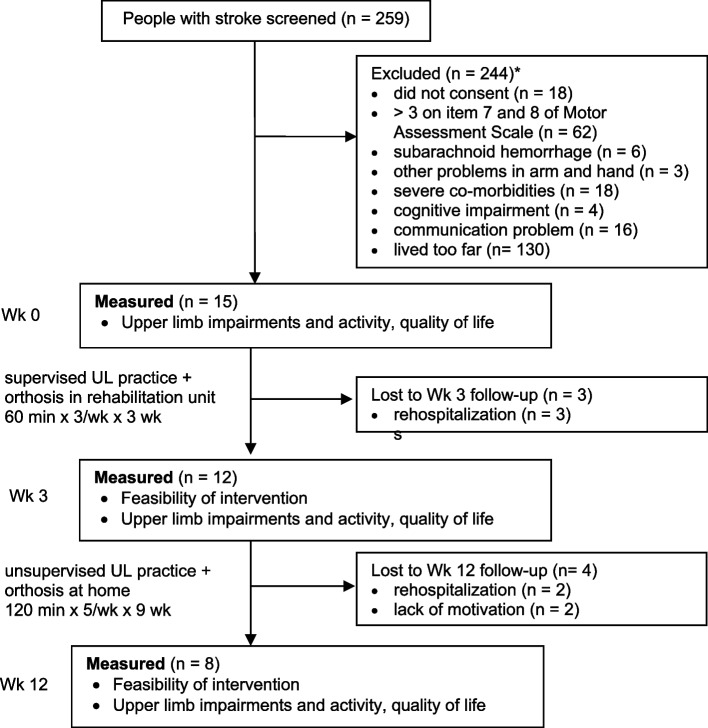
Flow diagram of participants through the study. * Patients may have been excluded for more than one reason

Before progressing to a randomized trial, a single-group, pre-post intervention study was carried out (Fig. 1) since the aim was to determine feasibility of the intervention rather than efficacy. Considering the limited availability of useful information in our specific context, population, and intervention, this initial study was focused entirely on feasibility outcomes. A second pilot study, with full randomization, will be implemented with the insights gained from the current study. Adult stroke survivors with reduced arm and hand function were recruited from a community rehabilitation unit in Norway. Measures were collected at Week 0 (baseline), at Week 3 (after supervised training in the unit), and at Week 12 (after unsupervised training at home) by an independent measurer not involved in the intervention. Ethical approval was obtained from the regional ethics committee in Norway (2017/191). All participants were provided with written information about the study and gave written informed consent. The study was registered with the ClinicalTrials.gov identifier NCT03396939.

### Participants

The inclusion criteria were: admitted with a diagnosis of stroke to the community rehabilitation unit, age > 18 years and able to consent, reduced arm and hand function determined by a score ≤ 3 on the Motor Assessment Scale (MAS) Items 7 (hand movements) and 8 (advanced hand functions) [15]. Potential participants were excluded if they had language and/or cognitive impairments that precluded them from following instructions (defined as Montreal Cognitive Assessment ≤ 20 [16], Goodglass-Kaplan Aphasia Severity Rating Scale < 2 [17]), severe comorbidities or other health conditions that precluded the person from undergoing upper limb rehabilitation. Eligible participants were enrolled if they resided within 30 km of the community unit. We planned to recruit 30 participants as this is considered an adequate number to assess feasibility [18].

### Intervention

All training sessions were performed using a dynamic orthosis. A commercial dynamic hand orthosis (Saeboglove ®, Saebo Inc, Charlotte, NC) was used. It consists of a soft Lycra glove, a spiraled forearm splint and an extension system with five sized tensioners. The glove is designed to maintain the wrist in neutral and assist finger and thumb extension following a grasping motion via tensioners at the interphalangeal joints. Therapist and user can customize the tension at joints. The intervention was performed individually and lasted for 12 weeks and included training both at the community rehabilitation unit and at home. Therapist-supervised upper limb practice was delivered in nine 60-min sessions over 3 weeks at the community rehabilitation unit. These sessions could be divided into smaller sessions but were to total 60 min. This was followed by 120 min of unsupervised home practice five times a week over 9 weeks. That is, participants were expected to complete 111 h of training. The structure of the program was standardized but individualized in content to account for the difference in goals, barriers, and solutions between participants. At the community rehabilitation unit, upper limb practice included 10 min of gross motor training (e.g. reaching to the opposite shoulder, hip, top and back of head with the hand), 10 min of fine motor training (e.g. pinch, grip, writing, turning pages, handling objects), 10 min of strength training, and 30 min of ADL (e.g. washing up, folding laundry, sweeping floor). One phone call was made in the middle of the 9-week home training to follow up on progress.

### Measurement of feasibility

We determined the feasibility of recruitment, intervention, and measurement. Feasibility of recruitment was determined as the proportion of eligible/enrolled (≥ 50% to be deemed adequate) and enrolled/retained (≥ 85% to be deemed adequate) participants from the population of people having had a stroke admitted to the rehabilitation unit.

Feasibility of the intervention was determined as adherence, acceptability, and safety. Adherence of the participant to the intervention and average time taken for each session of training were recorded using an exercise log (≥ 80% to be deemed adequate). Acceptability of the intervention was determined from a semi-structured interview involving questions about their experiences of wearing the dynamic orthosis and willingness to recommend the program to others. An interview guide was prepared in advance and used as a checklist to ensure that the participants addressed the topic of interest. Each interview was recorded with the help of a voice recorder after getting the participant’s approval. Shortly after each interview, the data were transcribed into written form. Participants rated their satisfaction from 1 to 5 (strongly dissatisfied to strongly satisfied) regarding the program as a whole, type of exercises (i.e., fine motor, combined and strengthening exercises), dose and duration of intervention (≥ 50% scoring satisfied to be deemed adequate). Safety was determined by recording events such as fatigue, non-injurious falls, injurious falls, hospitalization, and death related to the intervention (≤ 1 per participant to be deemed adequate).

Feasibility of measurement was determined as the time taken to collect outcomes (≤ 1 h to be deemed adequate), proportion of participants where all measures were collected (≥ 85% to be deemed adequate) and the appropriateness of outcome measurement tools. Outcomes were measured using valid and reliable measures of upper limb activity, impairment and health-related quality of life. Upper limb activity was measured using the Action Research Arm Test (ARAT) [19], the Motor Assessment Scale for Stroke (MAS) [15, 20] and the Nine-hole Peg Test (9HPT) [21]. ARAT (0–57) consists of 19 items rated on a four-point ordinal scale (0–3). The reliability and validity of the Norwegian translation of the scale have been established [22]. Scores of 9HPT were reported as pegs/s. Three items of the MAS (0–6) – Item 6 for upper arm function, Item 7 for hand movements and Item 8 for advanced hand function – were collected in this study. Measures of upper limb impairment following stroke were maximal hand grip strength using a Jamar dynamometer (kg), modified Ashworth Scale (0–4) to assess spasticity of the hand [23, 24], and Fugl-Meyer Assessment Upper Extremity (FMA-UE) to assess the degree of synergy development (0–66) [25, 26]. Overall health status was measured using the visual analogue scale from the EuroQual-5D (EQ-5D) (0–100) [27].

### Data analysis

The feasibility and baseline characteristics of participants are presented as mean (SD) and/or median (IQR), or number (%). For clinical outcomes, the within-group comparisons between measurement points are presented as the mean difference (95% confidence interval) between Week 0 and Week 3 to reflect training at the community rehabilitation center, and Week 0 and Week 12 to reflect training at home. We used a per-protocol analysis approach. Analyses were performed using STATA version 16 for Mac (STATA Corp., Texas, USA).

## Results

### Characteristics of participants

Fifteen individuals with stroke aged 67 (SD 15) years and 145 (SD 152) days after stroke participated in the study (Table 1). They were moderate-to-severely disabled at the activity and impairment level with an ARAT score of 17/57 (SD 13) and FMA-UE score of 32/66 (SD 13). On average, participants received 2.1 supervised hours (SD 0.4) of 3.1 (SD 0.7) different therapies in addition to the experimental intervention at the rehabilitation center. At three weeks, three participants had withdrawn from the study due to rehospitalization. At 12 weeks, a further four participants had withdrawn; two due to rehospitalization, and two due to lack of motivation to complete the intervention. The flow of participants through the study is shown in Fig. 1.

**Table 1:** Characteristics of participants at baseline

Characteristic	*n* = 15
Age *(yr)*, mean (SD)	67 (15)
Sex,* n* males (%)	8 (53)
Time since stroke (wk), mean (SD)	21 (22)
Ischemic stroke, *n* (%)	8 (53)
Dominant hand, *n* right (%)	12 (80)
Side of hemiplegia, *n* right (%)	6 (40)
Medical conditions *n* (%)	
Hypertension	8 (53)
Atrial fibrillation	1 (7)
Diabetes mellitus	2 (13)
Other	13 (87)
Medications, mean (SD)	5.6 (2.4)
Marital status, *n* de facto & married (%)	7 (47)
Residence, *n* house (%)	8 (53)
Occupation, *n* retired (%)	10 (67)

### Feasibility

#### Recruitment

A total of 259 patients after stroke were screened for eligibility for the study between February 2018 and May 2019 (Fig. 1). 15 (46%) of the 33 eligible were enrolled in the study. The study was terminated early before reaching the intended 30 participants because of slow progress and large number of patients living too far from the recruitment center. Eight (53%) of those enrolled completed the 12-week intervention. Five participants were lost to follow up because they were rehospitalized.

#### Intervention

All eight participants who completed the intervention appeared to meet the prescribed dose and intensity. However, adherence of training could not be confirmed as it was not consistently written in the exercise log. In terms of acceptability, of those who completed the 12-week intervention, 7 (88%) were either satisfied or very satisfied with the dynamic hand orthosis. Some of them extended the use beyond training into their everyday life as an aid. As cited by one participant.

*“I can't straighten my fingers and my hand hangs down. The splint helps me get my hand straight into the grip position, and the tensioners help me get my fingers out when I grab things. I use the tool a lot in my daily life. Some days I wear it all day. When I take it off after using it for a while, my hand is soft and fine. I get to use my affected hand and thus my entire body. If I only use one hand, I have to compensate a lot with the rest of the body.” (Participant 4)*

Six (75%) were either satisfied or very satisfied with the program as a whole and 7 (88%) were willing to recommend it to others in the same condition. Fifty to 88% were either satisfied or very satisfied in terms of type of exercises and dose of the intervention. and duration of intervention (Table 2). Most of the participants agreed that the glove helped them to stretch fingers out, make grip and release objects.

*“Saeboglove is good as it is a simple tool. The tensioners last a long time, the splint straightens the wrist for you so that the hand is in the grip position. When you do not use the hand, the fingers are straightened automatically. It can be used as a training tool and not least as an aid in daily life.” (Participant 9)*

**Table 2:** Feasibility of intervention in terms of satisfaction and safety (*n* = 15)

Feasibility	Week 0–3		Week 4–12
*Acceptability*, n satisfied and very satisfied (%)			
• Using the dynamic hand orthosis	7 (58)		7 (88)
• Willing to recommend the orthosis to others, n agree (%)	11 (92)		7 (88)
• Program as a whole	7 (58)		6 (75)
• Type of exercises			
General	8 (67)		4 (50)
Fine motor	8 (67)		5 (63)
Combined	7 (58)		5 (63)
Strength	7 (58)		6 (75)
*Safety,* n			
Fatigue	0		0
Non-injurious falls	0		0
Injurious falls	0		0
Death	0		0
Rehospitalization			
Pneumonia	1		1
Cancer	1		0
Urinary tract infection	1		1

However, some expressed difficulties in mounting the glove without assistance of others. For example, one participant said,

*“Yes, I would recommend this to others in the same situation. However, the challenge is to get it on if you have a clenched hand. I had to train for a long time to loosen my hand so I could use the Saeboglove. One should be able to get it on without the help of others.” (Participant 11)*

In terms of safety, no fatigue, non-injurious falls, injurious falls or deaths were reported throughout the study. Five participants were rehospitalized, but none of these events was directly related to the intervention (Table 2).

#### Measurement

Duration used to collect measures ranged from 45 to 70 min depending on the disability level of participants. Clinical measures were collected for all participants at baseline and in all those who completed the intervention at Week 3 and Week 12.

The clinical outcomes for the 8 participants who completed the study are presented in Table 3. At 3 weeks, participants improved by 5 out of 66 points (95% CI 1 to 9) on FMA-UE compared with baseline. At 12 weeks, participants had improved by 7 points out of 57 (95% CI 2 to 13) on ARAT and by 8 points out of 66 (95% CI 0 to 15) on FMA-UE compared with baseline.

**Table 3:** Mean (SD) and median (IQR) of outcomes for each time and mean (95% CI) difference between times for participants who completed 12 weeks of training (*n* = 8)

Outcome	Times (*n* = 8)			Difference between times	
	Week 0	Week 3	Week 12	Week 3 minus Week 0	Week 12 minus Week 0
Action Research Arm Test (0–57)	14 (13) 9 (4–27)	18 (13) 16 (8–30)	21 (16) 15 (9–37)	4 (-2 to 10)	7 (2 to13)
Nine-hole Peg Test (pegs/s)	0.04 (0.07) 0 (0–0.03)	0.04 (0.08) 0 (0–0.03)	0.07 (0.12) 0 (0–0.10)	0.01 (-0.03 to 0.04)	0.03 (-0.01 to 0.07)
Motor Assessment Scale (0–6)					
Item 6	1.1 (1.6) (0.0–2)	1.1 (1.6) 0 (0.0–2.5)	2.0 (2.2) 2 (0–4)	0.0 (-1.6 to 1.6)	0.9 (-0.3 to 2.1)
Item 7	0.6 (1.1) 0 (0–1)	1.3 (2.2) 0 (0–2)	1.3 (2.2) 0 (0–2)	0.6 (-0.4 to 1.6)	0.6 (-0.4 to 1.6)
Item 8	0.6 (0.9) 0 (0–2)	0.8 (0.9) 1 (0–2)	0.9 (0.8) 1 (0–2)	0.1 (-0.2 to 0.4)	0.3 (-0.3 to 0.8)
Grip strength (kg*)*	5.9 (6.0)	7.9 (7.2)	8.3 (7.6)	2.0 (-0.4 to 4.4)	2.4 (-0.9 to 5.6)
Modified Ashworth Scale (0–4) finger flexion	1.6 (1.4) 2 (0–3)	1.1 (1.1) 1 (0–2)	1.4 (1.4) 1 (0- 3)	-0.5 (-1.4 to 0.4)	-0.3 (-1.7 to 1.2)
Fugl-Meyer Assessment-Upper Extremity (0–66)	29 (11) 27 (22–38)	34 (14) 31 (25–48)	37 (17) 34 (23–53)	5 (1 to 9)	8 (0 to 15)
EQ-5D visual analog scale (0–100)	48 (23)	52 (23)	55 (23)	4 (-14 to 23)	7 (-5 to 19)

## Discussion

Incorporating the dynamic hand orthosis – Saeboglove – into an upper limb program appears to be feasible in people with reduced arm and hand function after stroke in terms of acceptability and safety. The majority of those who completed the 12-week intervention were highly satisfied with the program and there were no adverse events. Statistically and clinically significant improvements were achieved on the ARAT and FMA-UE, suggesting that this combination has a potential to improve both upper limb activity and impairment.

In terms of recruitment, 94% were excluded after screening with the most common reason for exclusion being that because the person lived too far from the rehabilitation unit to allow for follow-up. This suggests that a future trial should be multi-site or perhaps the intervention could be wholly home-based. Furthermore, recruitment of eligible participants was low, and the study was terminated without achieving the intended sample size. Although 47% were lost to follow-up by 12 weeks, only 15% was due to unwillingness to continue with the intervention. Factors that impede eligible participants to enter study (e.g., strict inclusion and exclusion criteria, complex and lengthy intervention) should be carefully considered and certain incentives (e.g., compensating travelling expenses, providing new training opportunities) may be given to participants at study completion to prevent early withdrawal from the trial.

In terms of the intervention, although most of the participants found the intervention acceptable, there were several challenges. During the home-based practice, people with severe disability had difficulty donning the glove without assistance. Our study involved people who were severely disabled after stroke who scored < 50% of maximum score of ARAT and could not move a peg on the 9-HPT at baseline. It may be appropriate to include a minimum level of upper limb function as criteria for eligibility in future. Furthermore, we were unable to confirm the adherence to the home protocol and objective monitoring of participants activity is recommended in future.

In terms of measurement, the length of time taken to complete the outcome measurements could be decreased by collecting only one measure under each domain of upper limb activity, impairment, and participation. For example, the ARAT reflects upper limb activity while the FMA-UE reflects impairment as suggested by the Stroke Recovery Round Table [28].

Our clinical results are in line with Franck et al. who found that stroke survivors could benefit from hand training with a dynamic orthosis [29] as measured by the ARAT. Nonetheless, the lack of a control group poses a challenge in drawing meaningful conclusions from our present findings. The early supervised sessions allowed the participants to learn how to participate in a task-oriented exercise program with a severely disabled upper limb. The dynamic orthosis enabled them to use their affected upper limb and generated a sense of self-efficacy which resulted in high satisfaction. The acceptability of similar technology-assisted, home-based interventions has been reported in several studies [29–32]. Such intervention may be a future solution for many stroke survivors as self-directed home-based practice are now more commonly prescribed.

However, limitations in the present study can help to inform future trials. For example, our participants varied greatly in terms of time since stroke which ranged from 34 to 613 days. Motor improvement appears to begin to plateau at about 3 months after stroke [28, 33]. Therefore, changing the eligibility criteria to include people within 3 months of stroke will enable implementation of high intensity, task-oriented training early after stroke which has been shown to be important in improving motor activity [34].

In conclusion, upper limb practice with a dynamic hand orthosis appears to be feasible in terms of acceptability and safety and has the potential to improve arm and hand function in people after stroke. Recruitment and measurement issues should be addressed in future studies. The magnitude of the clinical outcomes suggests that the intervention has a potential to improve both upper limb activity and impairment. Further investigation in disabled people early after stroke is needed, and this study provides useful information for the design of a randomized trial.

## Data Availability

The datasets used during the current study are available from the corresponding author on reasonable request.

## Abbreviations

ADL: Activities of daily living
MAS: Motor Assessment Scale
ARAT: Action Research Arm Test
FMA-UE: Fugl-Meyer Assessment Upper Extremity
9HPT: Nine-hole Peg Test
EQ-5D: EuroQual-5D
SD: Standard deviation
95% CI: 95% Confidence interval
